# Construction of a bacterial artificial chromosome library from the spikemoss *Selaginella moellendorffii*: a new resource for plant comparative genomics

**DOI:** 10.1186/1471-2229-5-10

**Published:** 2005-06-14

**Authors:** Wenming Wang, Milos Tanurdzic, Meizhong Luo, Nicholas Sisneros, Hye Ran Kim, Jing-Ke Weng, Dave Kudrna, Christopher Mueller, K Arumuganathan, John Carlson, Clint Chapple, Claude de Pamphilis, Dina Mandoli, Jeff Tomkins, Rod A Wing, Jo Ann Banks

**Affiliations:** 1 Arizona Genomics Institute, Department of Plant Sciences, University of Arizona, Tucson, AZ 85721, USA; 2 Department of Botany and Plant Pathology, Purdue University, West Lafayette, IN 47907, USA; 3 Department of Biochemistry, Purdue University, West Lafayette, IN 47907, USA; 4 Benaroya Research Institute at Virginia Mason, 1201 Ninth Avenue, Seattle, WA 98101, USA; 5 Department of Biology and Huck Institutes of Life Sciences, Penn State University, University Park, PA 16802, USA; 6 Department of Biology and Center for Developmental Biology, University of Washington, Seattle, WA 98195, USA; 7 Department of Genetics, Biochemistry and Life Science Studies, Clemson University, Clemson, SC 29634, USA; 8 Cold Spring Harbor Laboratory, Cold Spring Harbor, NY 11724, USA; 9 Center for Biosystems Research, University of Maryland Biotechnology Institute, College Park, MD 20742, USA

## Abstract

**Background:**

The lycophytes are an ancient lineage of vascular plants that diverged from the seed plant lineage about 400 Myr ago. Although the lycophytes occupy an important phylogenetic position for understanding the evolution of plants and their genomes, no genomic resources exist for this group of plants.

**Results:**

Here we describe the construction of a large-insert bacterial artificial chromosome (BAC) library from the lycophyte *Selaginella moellendorffii*. Based on cell flow cytometry, this species has the smallest genome size among the different lycophytes tested, including *Huperzia lucidula*, *Diphaiastrum digita*, *Isoetes engelmanii *and *S. kraussiana*. The arrayed BAC library consists of 9126 clones; the average insert size is estimated to be 122 kb. Inserts of chloroplast origin account for 2.3% of the clones. The BAC library contains an estimated ten genome-equivalents based on DNA hybridizations using five single-copy and two duplicated *S. moellendorffii *genes as probes.

**Conclusion:**

The *S. moellenforffii *BAC library, the first to be constructed from a lycophyte, will be useful to the scientific community as a resource for comparative plant genomics and evolution.

## Background

The lycophytes (class Lycopsida) are an ancient group of vascular plants that dominated the earth's flora during the Carboniferous period. The three orders of lycophytes that remain from this period include the homosporous Lycopodiales, the heterosporous Selaginellales and the heterosporous Isoetales. All of these plants are distinguishable from ferns and flowering plants by the presence of microphylls (as opposed to euphylls), the absence of leaf gaps and the absence of lateral roots. In common with ferns but not flowering plants, all lycophytes produce free-living spores, an independent gametophyte generation and non-integumented sporangia. Based upon the fossil record, the lycophytes are thought to have emerged during the early Devonian about 400 Myr ago prior to the evolution of leaves and roots in vascular plants [1,2]. Based on recent DNA-based phylogenetic analyses, the Lycopsida clade is monophyletic and sister to the fern/seed plant, or euphyllophyte, clade [3]. As representatives of the earliest and still-surviving vascular plant lineage, the lycophytes are an important group of plants for providing insights into the early evolution of land plants.

While genomic resources are available for many species of flowering plants, including the sequences of the *Arabidopsis thaliana *[4], rice [5,6] and poplar [7] genomes, very few resources exist for plants other than angiosperms. A draft genome sequence is available for *Chlamydomonas reinhardtii *[8], a chlorophytic green alga that is a distant relative of the charophytic algal group that gave rise to land plants [9]. The huge phylogenetic gap between the characterized genomes of *Chlamydomonas reinhardtii *and flowering plants greatly limits our ability to study how important features in plants originated and diversified at a genetic level. To help fill this gap, the genome sizes of several species of lycophytes were determined by cell flow cytometry in order to identify a lycophyte with a relatively small genome. Of those surveyed, the spikemoss *Selaginella moellendorffii *was found to have the smallest, with a nuclear genome size less than 127 Mbp. Here we describe the construction and characterization of a large insert BAC library for this species.

## Results and discussion

### Genome size estimates

The approximate genome sizes of several lycophyte species were determined by cell flow cytometry using as an internal standard the nuclei of other plants or cells with genomes of known sizes (Table 1). The homosporous *Huperzia lucidula *and *Diphaiastrum digita *(Lycopodiales) have the largest genomes of those surveyed, estimated to be 5585 and 2670 Mbp/1C, respectively. *Isoetes engelmanii *(Isoetales), a heterosporous lycophyte, has a genome size of 1710 Mbp/1C. The heterosporous *Selaginella moellendorffii *(Selaginellales) has the smallest genome of those surveyed, between 88 and 127 Mbp/1C. The three estimates of *S. moellendorffii *genome size (Table 1) vary depending on species used as the internal standard. We also confirmed that the genome size of *S. moellendorffii *is smaller than that of *S. kraussiana *(Table 1), which was previously reported to have a genome size ranging from 0.32–0.72 pg/2C, or 157–320 Mbp/1C [10]. The decision to construct a BAC library from the heterosporous *S. moellendorffii *was largely based on its having the smallest genome size of all lycophyte accessions examined here or reported previously.

### BAC library construction and characterization

**Figure 1:**
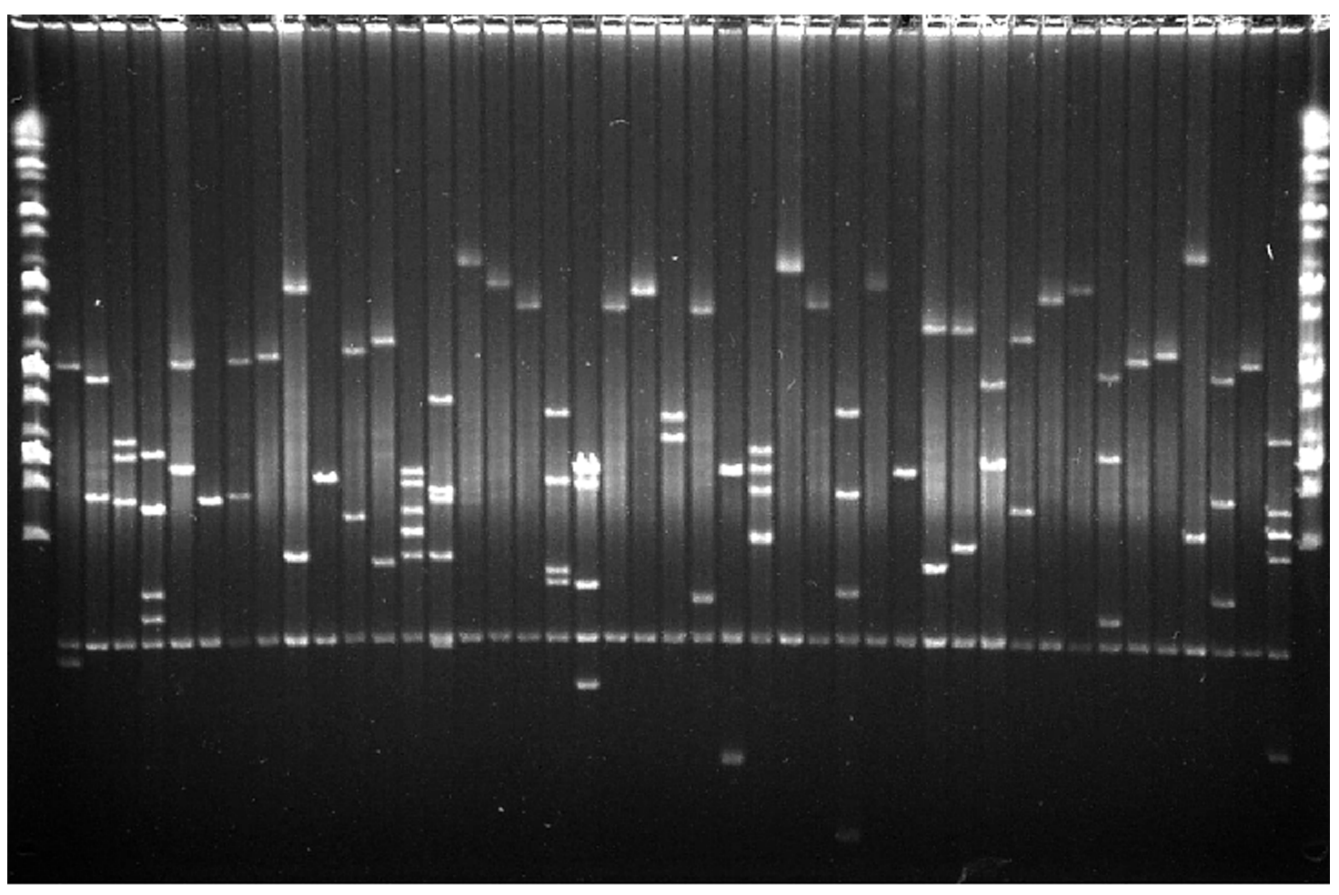
*Not *I digests of BAC DNA isolated from 43 random BAC clones from the *S. moellendorffii *BAC library. The vector contains two *Not *I sites that flank the *Hind *III cloning sites. The vector is present in all samples with the exception of the first and last lanes, which contain the DNA marker PFG midrange I (New England Biolabs, Beverly, MA).

**Figure 2:**
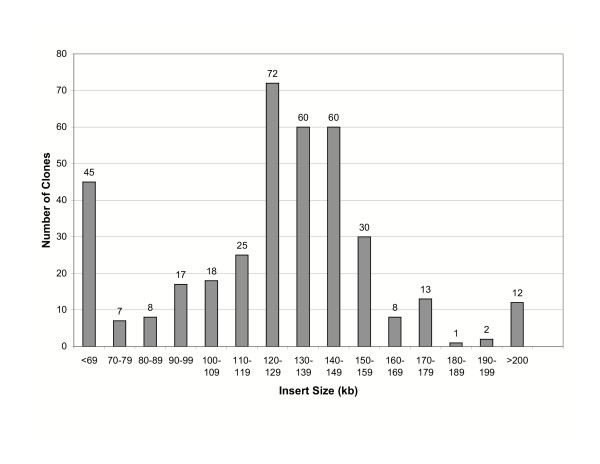
The distribution of BAC insert sizes from 410 randomly chosen BAC clones.

In constructing the BAC library, nuclear DNA was isolated from the growing tips of *S. mollendorffii *sporophytes, partially digested with *Hind *III and the library constructed and processed essentially according to Luo and Wing [11] as described in the Methods section. A total of 9126 BAC clones were picked, arrayed and processed for long-term storage. To estimate the insert sizes of this library, BAC DNAs were prepared from 410 randomly selected clones, digested with *Not *I and size-separated by pulse field gel electrophoresis. The *Not *I restriction patterns of 43 randomly selected clones are illustrated in Figure 1. Of the 410 BAC DNAs prepared, 23 yielded no DNA and 9 lacked an insert. Of the remaining 378 clones with inserts, the inserts ranged in size from 9 to 292 kb, with 75% having inserts sizes 90–159 kb and 84% with inserts greater than 90 kb. The average insert size of the clones with inserts was 122 +/- 44 kb (SD). The distribution of insert sizes is shown in Figure 2.

To estimate the extent of chloroplast and mitochondrial DNA contamination of the BAC library, the arrayed library was probed with two *S. moellendorffi *DNA fragments that contain either chloroplast- or mitochondrial-encoded genes (Table 1). The *S. moellendorffii *DNA fragment containing the chloroplast encoded ribosomal proteins S8, L2 and S19 hybridized to 207 BAC clones. The *S. moellendorffii *DNA fragment containing the mitochondria-encoded *NADH DEHYDROGENASE SUBUNIT 5 *gene did not hybridize to any BAC clones but did hybridize to itself (data not shown). It is unlikely that either fragment is of nuclear origin given that these genes are encoded by organelles in every plant species studied so far. The results of these hybridizations demonstrate that a very small proportion (2.3%) of the BAC inserts are of chloroplast origin. This is within the expected range for organellar DNA contamination in large insert DNA libraries [12]. The inability to detect clones that hybridize to mitochondrial DNA might reflect a mitochondrial genome that is small enough to be efficiently removed from the nuclear DNA preparation. However, the sizes of mitochondrial genomes in the lycophytes have yet to be reported.

**Figure 3:**
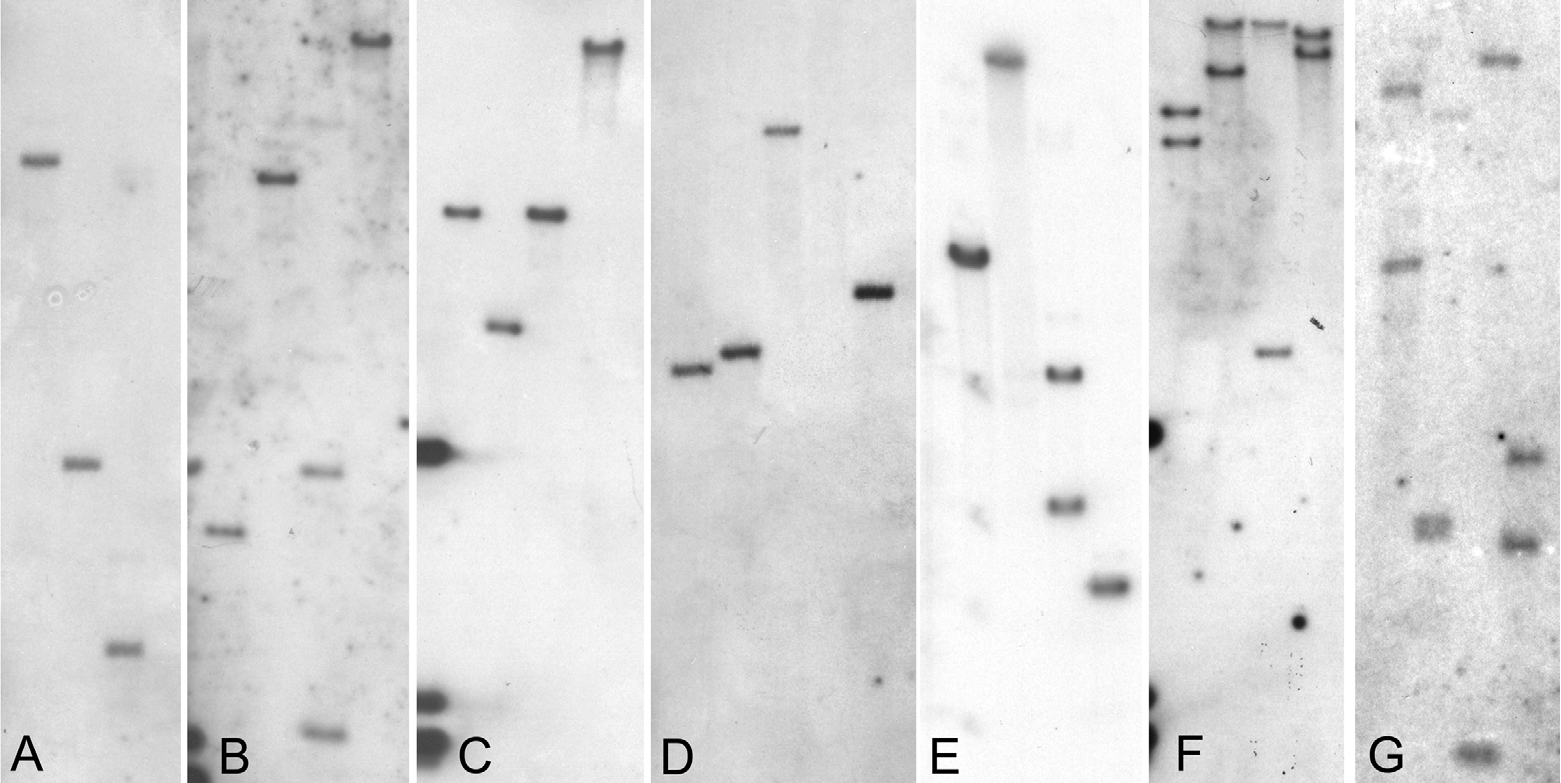
Results of DNA blots to determine the gene copy number of various DNA fragments in the *S. moellendorffii *genome. DNA from *S. moellendorffii *was digested with *EcoR *V, *EcoR *I, *Nco *I or *Hind *III and probed with the *SmSNT *fragment (panel A), the *SmSHR *fragment (panel B), the *SmZNF *fragment (panel C), the *SmChlH *fragment (panel D), the *SmGAI *fragment (panel E), the *SmOTP *cDNA (panel F), and the *SmCYP98 *cDNA (panel G). The *EcoR *V digest is absent in panel A. The left-most lanes in panels B, C and F contain molecular weight markers.

To determine the number of genome equivalents represented by the BAC library, the arrayed library was probed with the seven *S. moellendorffii *genes listed in Table 2. All seven genes potentially encode proteins that are at least 43% identical in amino acid sequence to similar genes in angiosperms. The two genes homologous to *GIBBERELLIN INSENSITIVE *(*SmGAI*) and *CYTOCHROME P450 98 *(*SmCYP98*) were obtained by RT-PCR. The gene homologous to *OXYANION TRANSLOCATION PROTEIN *(*SmOTP*) was identified from an EST library (C. Chapple, unpub. obs.), while the genes homologous to the *SHORTROOT *(*SmSHR*), *MAGNESIUM CHELATASE SUBUNIT H *(*SmChlH*), *ZINC-FINGER *(*SmZNF*) and *SYNTAXIN *(*SmSNT*) gene fragments used as probes were identified from the sequences generated from the ends a sheared *S. moellendorffii *genomic library (J. Banks, unpub. obs.). Based upon the genomic DNA hybridization results shown in Figure 3, these genes are present in either one or two copies in the genome. The number of BAC clones that hybridized to the single-copy genes ranged from 7–12, and to about twice that number of clones (16 and 23) using the two duplicated genes as probes (Table 2). Based on the results of these experiments, the estimated number of genome equivalents represented by the BAC library is ~10. Assuming that one nuclear genome equivalent is represented by 871 BAC clones with nuclear DNA inserts and each BAC insert is 122 kb, we estimate that the *S. moellendorffii *genome is 106 Mbp. This estimate coincides well with the estimated size of the nuclear genome of *S. moellendorffii *based upon cell flow cytometry.

To our knowledge, this is the first Lycopsida BAC library constructed. A BAC library was recently published for *Physcomitrella patens *[13], a moss that has become a popular model system for functional genomics, biochemistry, evolutionary and developmental genetic studies (reviewed by [14]). As a representative of the early branching vascular plants, the *S. moellendorffii *library described here will link genomic resources from algae (*Chlamydomonas reinhardtii*) and moss (*P. patens*) to seed plants, including important crop species. The library also will be a useful resource for readily identifying genes that are involved in developmental, physiological and biochemical processes in the lycophytes and provide an important tool for the study of plant evolution. The nuclear genome of *S. moellendorffi *is currently being sequenced by the Department of Energy Joint Genome Institute using a shotgun sequencing approach [15]. The BAC library described here is currently available to the scientific community through the Arizona Genomics Institute [16].

## Conclusion

We have shown that the lycophyte *S. moellendorffii *has a very small genome size, as small or smaller than that of *Arabidopsis thaliana *based on cell flow cytometry, and have constructed from this species a large insert BAC library that contains about 10 genome equivalents and has an average insert size of 122 kb.

## Methods

### Plant material

*Selaginella moellendorffii *plants were obtained from Plant Delights Nursery, Inc., Raleigh, NC. *Huperzia lucidula *plants were obtained from Carolina Biological Supply Company (Burlington, NC; referred to there as *Lycopodium lucidulum*). *Diphaiastrum digita *plants were obtained from Gar Rothwell (Ohio University, Athens, OH). *Isoetes engelmannii *plants were obtained from Gerald Gastony (Indiana University, Bloomington, IN). Once obtained, all plants were grown in a local greenhouse under 50% shade cloth.

### Nuclear DNA content determination

The procedure used to analyze nuclear DNA content in plant cells was modified from Arumuganathan and Earle [17]. *Glycine max*, *Oryza sativa *cv Nipponbare or *Arabidopsis thaliana *or chicken red blood cell nuclei were used as internal standards. For flow cytometric analysis, 50 mg of fresh leaf tissue was placed on ice in a sterile 35 × 10 mm plastic petri dish. The tissue was sliced into 0.25 mm to 1 mm segments in a solution containing 10 mM MgSO_4_, 50 mM KCl, 5 mM Hepes, pH 8.0, 3 mM dithiothreitol, 0.1 mg ml^-1 ^propidium iodide, 1.5 mg ml^-1 ^DNase free RNase (Rhoche, Indianapolis, IN) and 0.25% (v/v) Triton X-100. The suspended nuclei were filtered through 30 μm nylon mesh and incubated at 37 C for 30 min before flow cytometric analysis. Suspensions of sample nuclei were each spiked with a suspension of standard nuclei (prepared in above solution) and analyzed with a FAC calibur flow cytometer (Becton-Dickinson, San Jose, CA). For each measurement, the propidium iodide fluorescence area signals (FL2-A) from 1000 nuclei were collected and analyzed by CellQuest software (Becton-Dickinson, San Jose, CA) on a Macintosh computer. The mean position of the G0/G1 (Nuclei) peak of the sample and the internal standard were determined by CellQuest software. The mean nuclear DNA content of each plant sample, measured in pg, was based on 1000 scanned nuclei.

### BAC library construction

The growing tips (1 cm) of plants were harvested and flash frozen in liquid nitrogen prior to nuclei preparation. Purified nuclei were prepared according to Luo and Wing [11]. The embedding of nuclei, *Hind *III restriction enzyme digestion of DNA and the preparation of high molecular weight DNA fragments were performed according to Luo and Wing [11]. The *Hind *III cloning-ready single copy pIndigoBAC536 vector was prepared from the high copy pCUGIBAC1 plasmid as described by Luo et al. [18]. High molecular weight genomic DNA fragments were ligated to the vector and transformed into *E. coli *stain DH10B T1 phage-resistant cells (Invitrogen, Carlsbad, CA). Transformed colonies were picked and transferred into individual wells of 384 microtiter plates, grown and then stored at -80C. The BAC library was gridded onto 11.25 × 22.5 cm filters in high density, double spots and 4 × 4 patterns with a Genetix QB (Genetix, UK). To characterize the BAC inserts, BAC DNA samples were prepared with a Tomtec Quadra 96 model 320 (Tomtec, Hamden, CT) in a 96-well format, digested with *Not I*, separated on 1% agarose CHEF (Bio-Rad, Hercules, CA) gels at 5–15 sec linear ramp time, 6 V/cm, 14C in 0.5 × TBE buffer for 16 hours and stained with ethidium bromide.

### DNA hybridizations

Genomic DNA for gel blot analysis and PCR was isolated from *S. moellendorffii *plants using the Nucleon Phytopure kit (Amersham Biosciences, Piscataway, NJ). RNA was isolated using the RNeasy Plant Mini Kit (Qiagen, Valencia, CA); cDNA was synthesized and RT-PCR performed using the cMaster RTplusPCR System (Eppendorf, Westbury, NY). The 479 bp *SmGAI *cDNA fragment (GenBank accession AY874058) was initially obtained by RT-PCR using the primers 5'cayttyacigciaaycargci3' and 5' tcraaiarigcrctrtartartg3'. The 473 bp *SmCYP98 *cDNA fragment (GenBank accession AY843208) was obtained by RT-PCR using the primers 5'gtdgcvttcaacaacatwac3' and 5'ccatnccwgchgtgatcat3'. In all cases, y = c or t, r = a or g, d = a, g or t; v = a, c or g; w = a or t; n = a, t, g or c. All PCR products were cloned into the pGEM-T EASY vector (Promega, Madison, WI) and sequenced. Several genes used as probes were generated by PCR using genomic DNA as template. The 302 bp genomic *SmSHR *fragment (GenBank accession AY877259) was obtained using the primers 5'ggtggacctctcctctcctc3' and 5'atccaggtttgtagcgcttg3', the 254 bp genomic *SmZNF *fragment (GenBank accession AY877260) was obtained using the primers 5'gaggtcgtctccttgtcacc3' and 5'cggcgaaagtgtttcttgat3', the 395 bp genomic *SmChlH *fragment (GenBank accession AY877262) was obtained using the primers 5'ggatcgccttcatatccaaa3' and 5'aaactcgcggtcacagtctt3', and the 375 bp genomic *SmSNT *fragment (GenBank accession AY877261) was obtained using the primers 5'gatccaggccaagatgaaga3' and 5'tgccagtgaccgtgaagtag3'. The *SmOTP *gene (accession number AY877263) was identified from a *S. moellendorffii *EST library; the entire insert was used as a probe. The *S. moellendorffii *chloroplast (GenBank accession AY877264) and mitochondrial (GenBank accession AY877265) fragment sequences were identified from a partially sequenced, small-insert, sheared genomic library. For DNA blot hybridizations, each cloned insert was gel purified and labeled with ^32^P using the Megaprime DNA Labelling System (Amersham Biosciences, Piscataway, NJ). For DNA gel blots, 4 μg of *S. moellendorffii *genomic DNA was digested with restriction enzymes, fractionated and alkaline transferred to nylon membranes according to Sambrook and Russell [19]. All filters were hybridized at 65C in a solution containing 0.5 M phosphate buffer (pH 7.2), 7% (w/v) SDS, and 1 mM EDTA. All membranes were washed under stringent conditions (0.1X SSC, 0.1% SDS, 65C).

## Authors' contributions

JAB, JC, CC, CD, DM, JT and RAW participated in the design of this study. KA performed the cell flow cytometry genome size determination. ML and WW constructed the BAC library and DK, CM, HK, NS, ML, MT, WW and JW participated in library characterization. All authors have read and approved of this study.

## Figures and Tables

**Table 1:** Results of cell flow cytometry to determine the nuclear genome sizes of some lycophytes.

Sample species	Internal standard (pg/2C)	DNA content of sample (pg/2C); n^1^; SD	DNA content of sample species (Mbp/1C)^2^
*Huperzia lucidula*	Chick red blood cells (2.33^3^)	11.40; 4; 0.036	5586
*Diphaiastrum digita*	Chick red blood cells (2.33^3^)	5.45; 4; 0.017	2670
*Isoetes engelmanii*	Glycine max leaf (2.25^4^)	3.49; 4; 0.005	1710
*Selaginella moellendorffii*	*Arabidopsis thaliana *flower buds (0.36^5^) *Oryza sativa *leaf (1.0^6^) *Glycine max *leaf (2.25^4^)	0.18; 8; 0.009 0.25; 4; 0.25 0.26; 4; 0.006	88 123 127
*Selaginella kraussiana*	*Oryza sativa *leaf (1.0^6^) *Glycine max *leaf (2.25^4^)	0.43; 4; 0.006 0.49; 4; 0.003	211 240

^1^nuclei from sample and internal standard source plants or cells were prepared; n represents the number of times each nuclei preparation was sampled by cell flow cytometry.

^2^assumes that 1 pg = 980 Mbp

^3^Galbraith et al. [20]

^4^Bennett and Leitch [21]

^5^Bennett et al. [22]

^6^Bennett et al. [23]

**Table 2:** Results of DNA hybridizations to determine gene copy number and the number of genome equivalents represented in the arrayed BAC library.

Gene probe:	Homologous to following plant genes (e value; % amino acid identity)^1^:	Genome copy number:	Number of positive BAC clones from arrayed library:
*SmGAI *cDNA	*GIBBERELLIC ACID INSENSITIVE *(8e^-34^; 43)	1	14
*SmCYP98 *cDNA	*CYTOCHROME P450 98A3 *(1e^-40^; 66)	2	23
*SmOTP*	*OXYANION TRANSLOCATION PROTEIN *(1e^-36^; 53)	2	16
*SmSHR*	*SHORTROOT *(3e^-45^; 47)	1	7
*SmZNF*	*Zn-FINGER PROTEIN *(1e^-69^; 90)	1	7
*SmChlH*	*Mg CHELATASE SUBUNIT H *(1e^-130^; 85)	1	8
*SmSNT*	*SYNTAXIN *(9e^-55^; 54)	1	12
*S. moellendorffii *chloroplast fragment^2^	Contains: Ribosomal protein L22 (2e^-33^; 56) Ribosomal protein L2 (1e^-33^; 71) Ribosomal protein S19 (2e^-31^; 71)	ND	207
*S. moellendorffii *mitochondrial fragment^2^	Contains *NADH DEHYDROGENASE subunit 5 *(6e^-61^; 71)	ND	0

^1^Gene listed is based on results of blastx using the *S. moellendorffii *sequence as a query against the NR database and default parameters [24].

^2^sequence described is part of a genomic fragment <5 kb that was used as a probe.
